# Reliability Analysis of an Epileptic Seizure Detector Powered by an Energy Harvester

**DOI:** 10.3390/mi11010045

**Published:** 2019-12-30

**Authors:** Sunhee Kim, Suna Ju, Chang-Hyeon Ji

**Affiliations:** 1 Department of System Semiconductor Engineering, Sangmyung University, Cheonan-si 31066, Korea; 2 Department of Electronic and Electrical Engineering, Ewha Womans University, Seoul 03760, Korea; suna5290@gmail.com (S.J.); cji@ewha.ac.kr (C.-H.J.)

**Keywords:** energy harvesting, epilepsy, medical signal detection, performance estimation, and power supplies

## Abstract

Due to a limited lifetime of a battery, energy harvesters have been studied as alternative energy sources for implantable biomedical devices such as an implantable stimulator for epileptic seizure suppression. However, energy harvesters have weakness in providing stable power. We designed a neural recording circuit powered solely by a piezoelectric energy harvester, and applied its output to a seizure detector to analyze the reliability of the recorded signal. Performance of the seizure detector was evaluated. We found that the average time differences between with and without voltage variances were about 0.05 s under regular vibrations and about 0.07 s under irregular vibrations, respectively. The ratio of average true positive alarm period varied within about 0.02% under regular vibrations and 0.029% under irregular vibrations, respectively. The ratio of average false positive alarm period varied within about 0.004% under regular vibrations and 0.014% under irregular vibrations, respectively. This paper presents a reliability analysis of an epileptic seizure detector with a neural signal recording circuit powered by a piezoelectric energy harvester. The results showed that a supply voltage variance within ±10% could be acceptable for reliable operation of a seizure detector.

## 1. Introduction

Epilepsy is the fourth most common neurological disorder and affects approximately 65 million people around the world [1,2,3,4]. It is characterized by unpredictable and recurrent seizures [4,5]. Medication can control the majority of epileptic seizures and is almost the first therapy [4,5,6]. The 25%–30% of patients whose seizures cannot be controlled with anti-epileptic drugs may need other treatments such as seizure-trigger-area-removing surgery and neuro stimulation [4,5,6]. A neuro stimulation device for epileptic seizure is usually implanted within the skull under the scalp [5] and consists of one or two electrodes, a neural signal recording circuit, a seizure detector, and a stimulator. 

The implantable device has usually used a battery as its power source. The battery has to be replaced surgically after the battery is dead. This can lead to economic burden and psychological distress as well as physical pain [7]. Therefore, energy harvesting devices have attracted interest in the implant device field as alternative power sources because they can convert ambient energy into electrical energy [8,9,10,11,12]. Human-body motion based-harvesters, such as kinetic-energy or vibration-energy harvesters, are more useful [7]. Piezoelectric energy harvesting technique is one of the mechanical-to-electrical converting types. It has relatively high conversion efficiency, simple configuration, and high power density [13,14]. However, vibrations occurred at a natural environment, unlike an industrial environment, are distributed in low-frequency bands and vary unpredictably from time to time [15]. Therefore, energy harvesters including piezoelectric energy harvesters have been examined to find proper conditions to be used as stable or tolerable power sources. We studied conditions under which a targeted piezoelectric energy harvester can support stable power to a neural signal recording circuit. We analyzed the performance of an epileptic seizure detector that processes data from the neural recording circuit under the studied power conditions.

## 2. Materials and Methods 

We have estimated performance of a seizure detector when the seizure detector was connected to a neural recording circuit powered by a piezoelectric energy harvester. We could not test the harvester and circuits in vivo. We tested and analyzed power supply generated by the harvester and circuits powered from the generated power supply, particularly in the case that the power supply fluctuated. Then, we made their equivalent circuit models and simulated seizure data with the circuit models to estimate performance. Detailed description is as follows. 

### 2.1. Harvester and Circuits

**Figure 1:**
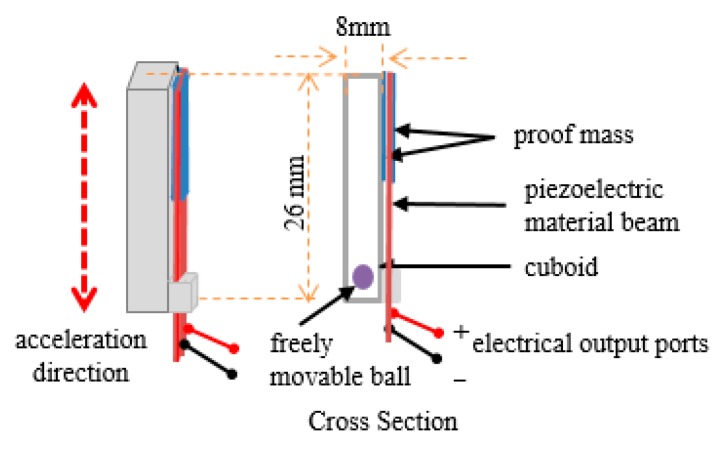
Diagram of the used frequency-up-converting impact-based piezoelectric energy harvester.

We have previously reported a frequency-up-converting impact-based piezoelectric energy harvester [16]. Figure 1 shows a diagram of the used frequency-up-converting impact-based piezoelectric energy harvester. It consisted of a 26 mm × 8 mm × 8 mm cuboid, a spherical ball with a radius of 2.5 mm inside the cuboid and a piezoceramic fiber-based macro fiber composite (MFC) beam. One end of the beam was fixed at the cuboid and the other end supported a proof mass like a cantilever. Two electrical ports worked as a reference signal and a generated voltage signal, respectively. 

When the harvester shook, the ball was bumped into the cuboid because the ball could move freely. This impact enabled the flexible MFC beam to be changed in shape and the MFC converted this force into an electrical charge. The generated voltage signal had higher frequency than the force applied to the harvester. It could increase the energy utilization by converting low-frequency mechanical vibrations such as human body movements into high-frequency electrical signals.

**Figure 2:**
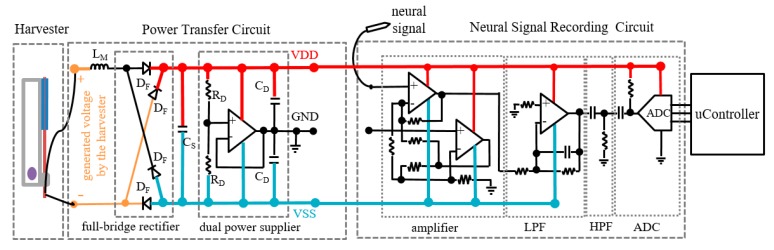
Power transfer circuits and neural signal recording circuits.

We composed power transfer circuits, which extracted power from the harvester and supplied stable power to load circuits, by using an inductor (L_M_), a full-bridge rectifier, a storage capacitor (C_S_), and a dual power supplier as shown in Figure 2. The inductor, L_M_, was used as a matching element because the piezoelectric harvester could generate voltage maximally at inductive loads [17]. The rectifier converted sparsely generated voltage signals to DC voltage, which was stored at the capacitor. The dual power supplier generated a positive power supply voltage (VDD) and a negative power supply voltage (VSS) for load circuits.

Neural signal recording circuits for an epileptic seizure detector consisted of an amplifier, a low pass filter (LPF), a high pass filter (HPF), and an analog-to-digital converter(ADC) as shown in Figure 2. An instrumentation amplifier was used at the first stage as an input buffer amplifier. The LPF and the HPF selected desired frequency components and removed DC offset. The ADC sampled processed signals and transferred them to a host. 

### 2.2. Test of the Harvester and the Circuits

SB120 Schottky barrier rectifiers (Vishay General Semiconductor, Malvern, PA, USA) were used for the full-bridge rectifier. AD8500 CMOS operational amplifiers (Analog Devices, Norwood, MA, USA) were used for the dual power supplier, the instrumentation amplifier, and the LPF. The matching inductor was 27 mH. An 8-bit ADC ADS7868 (Burr-Brown, Tucson, AZ, USA) was used. A field programmable gate array (FPGA) Starter including an Altera XC3S200 (Libertron, Seoul, Korea), a universal asynchronous receiver/transmitter (UART) connector was used to transfer the sampled data to a host. A test system was built by using a LDS V406 M4 shaker (Brüel and Kjær, Narum, Denmark), a 33220A waveform generator (Agilent, Santa Clara, CA, USA), a R300PLUS amplifier (Inter-M, Yangju, Korea), a SDS6062 digital oscilloscope (OWON, Zhangzhou, China), and an Agilent E3631A DC power supply (Agilent, Santa Clara, CA, USA). 

**Figure 3:**
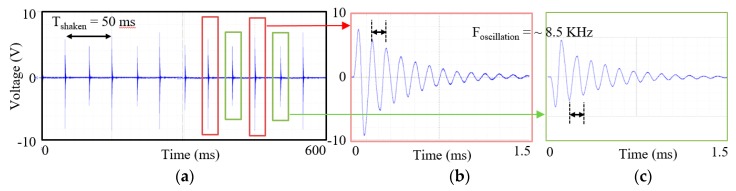
Measured open-circuit voltage at regular force: 20 Hz excitation with 3 g acceleration, (**a**) during 150 ms, (**b**) zoomed bottom-collision voltage during 1.5 ms, and (**c**) zoomed up-collision voltage during 1.5 ms.

Figure 3 shows measured open-circuit output voltage signals of the harvester when the shaker excited the harvester at acceleration of 3 g and frequency of 20 Hz in the same direction as shown in Figure 1. As shown in Figure 3a, the harvester generated voltage signals twice in one period of the applied low-frequency vibration because the freely movable ball impacted at both the bottom and top end in one cycle. Because expansion and contraction coefficients of the MFC as well as the collision forces at both sides were different, two different waveform signals were generated alternately as shown in Figure 3b,c. The generated signal oscillated around frequency of 8.5 KHz, attenuated almost exponentially after positive- negative peaks, and returned to zero.

**Figure 4:**
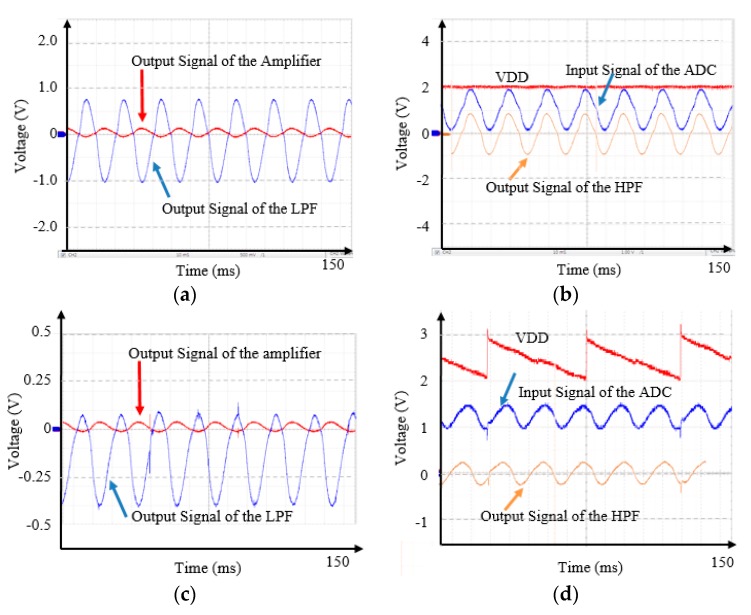
Measured data signals. (**a**) Output signals of the amplifier and the low pass filter (LPF) at Cs = 1 mF, (**b**) the positive supply voltage (VDD), the output signal of the high pass filter (HPF), and the input signal of the analog-to-digital converter(ADC) at Cs = 1 mF, (**c**) output signals of the amplifier and the LPF at Cs = 0.33 uF, and (**d**) the positive supply voltage VDD, the output signal of the HPF, and the input signal of the ADC at Cs = 0.33 uF.

To analyze the effects of the generated VDD on the neural signal recording circuits, we experimented as follows. Figure 4 shows measured signals of the circuits when the harvester shook at 20 Hz with 3 g and an input signal of the instrumentation amplifier was a 50 Hz sinusoidal voltage. The capacitance values of Cs in Figure 4a–d were 1 mF and 0.33 uF, respectively. The VDD of the circuits increased when an impact occurred, and then decreased until the next event. When the capacitance Cs was 1 mF, a ripple of the VDD was ±1.2% (Figure 4b). When the capacitance Cs was 0.33 uF, a ripple of the VDD was ±10% (Figure 4d), which we set as the worst case. Accordingly, the output voltage signals of the instrumentation amplifier, the LPF, and the HPF, and the input voltage signal of the ADC had distortions and spikes, especially when the VDD rose sharply. 

**Figure 5:**
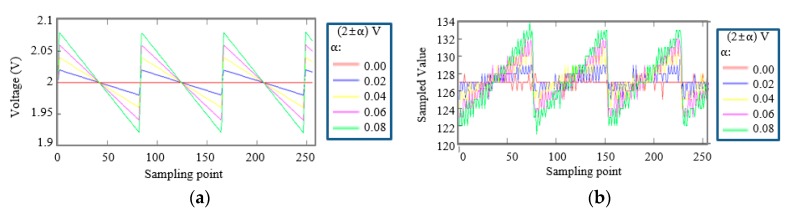
The sampled ADC data when an ADC was powered by a ramp signal. (**a**) Supply voltage signals with a (2 ± α) V ramp shape, and (**b**) the sampled ADC data.

We tested the ADC separately by using a commercial power supply instead of the harvester. Figure 5b shows the sampled data by an 8-bit ADC when the supply voltages of the ADC varied with slopes as shown in Figure 5a. The frequency of the supply voltages was 20 Hz (a 1 ms rising time and a 49 ms falling time). The magnitudes were (2 ± α) V, which α was 0, 0.02, 0.04, 0.06, and 0.08, respectively. The input signals of the ADC were fixed at 1 V, which was expected as their sampled data were 127 at a 2 V supply voltage. When the supply voltage decreased, input analog signals become larger relatively and vice versa. Therefore, the sampled data, with an average value of 127, were changed on the contrary to the supply voltages. In addition, even when the supply voltage was fixed at 2 V by a commercial power supply, the sampled ADC data had variations due to various noises.

**Figure 6:**
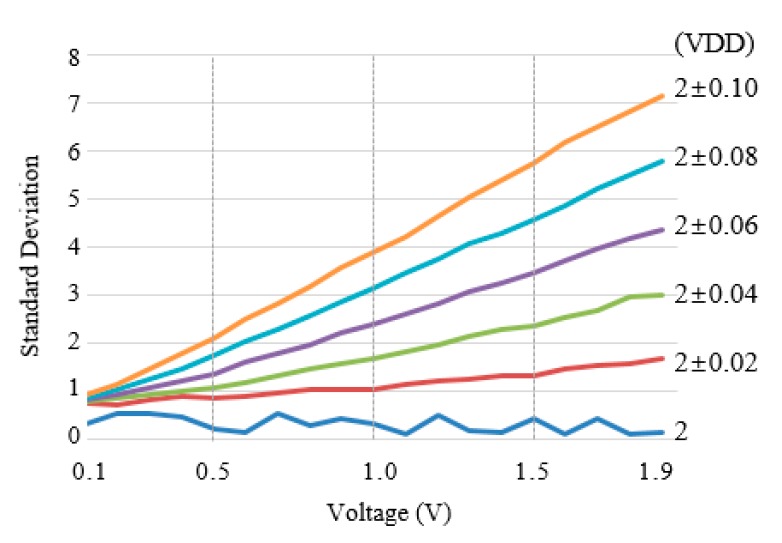
The standard deviation of the sampled ADC data according to VDDs and input voltage signals of the ADC in the range of 0.1 V to 1.9 V.

Figure 6 shows standard deviations of the sampled ADC data according to supply voltages and input signals of the ADC. The magnitudes of the supply voltages were (2 ± α) V, which α was 0, 0.02, 0.04, 0.06, 0.08, and 0.10, respectively. The amplitudes of the input signals were from 0.1 V to 1.9 V. When the variation of the supply voltage increased, the standard deviation of the sampled ADC data also increased. In addition, when the magnitude of the input signals increased, the standard deviation also increased.

In summary, the supply voltage generated by the harvester had some ripple and caused signal distortions and spikes. In addition, when processed signals was sampled at the ADC, they were modified according to their magnitude and the supply voltage magnitude at that time.

### 2.3. Seizure Detection Algorithm

We used a generic Osorio Frei algorithm (GOFA) [18] among known seizure detection algorithms. The GOFA is based on recorded data from intracranial electro- encephalography (icEEG) to extract the characteristic features of seizures: Energy and spectral analysis. The GOFA enables seizure detection by adjusting detection parameters according to energy and spectral elements. The GOFA and its modified algorithms usually consist of the following procedures [18,19,20,21].

#### 2.3.1. Filtering

A level-3 DAUB4 wavelet-based finite impulse response (FIR) filter extracts seizure-related frequency band components from icEEG that recorded at 240 Hz. If the recorded data are denoted as {*x_k_*|*k* = 1, 2, …}, the filtered data are given by

(1) $$y_{k}=\sum_{j=0}^{p-1}b_{j}x_{k-j}$$

where {*b*_0_, *b*_1_, ⋯ *b_p_*_−1_} and p are the coefficients and the order of the FIR filter, respectively.

#### 2.3.2. Calculating Foreground Sequences

The filtered data *y_k_* are squared and passed through a median filter. The median filter processes a moving window with the most recent 2 s of data and can separate short bursts such as certain artifacts or single spike. The resulting sequence is termed “foreground {*FG_k_*}” and given by

(2) $$FG_{k}=\mathrm{median}\left\{y_{k}^{2},y_{k-1}^{2},\ldots ,y_{k-\mathrm{OFG}+1}^{2}\right\}$$

where OFG = 480 is the order of the median filter for the foreground sequence.

#### 2.3.3. Calculating Background Sequences

To search the changes in the foreground, the foreground sequence is compared with a reference, which is called background {*BG_k_*}. The foreground sequence is sampled every 0.5 s and passed through another median filter.

(3) $$BG_{k}=\{\begin{array}{cc} \left(1-\lambda \right)\mathrm{median}\left\{FG_{k},FG_{k-s},\ldots ,FG_{k-\left(\mathrm{OBG}-1\right)s}\right\}+\left(\lambda \right)BG_{k-1}\;, & \mathrm{if}\;k=ns\\ BG_{k-1}, & if\;n\left(s-1\right)k\;ns \end{array}$$

where *n* = 0, 1, 2, ⋯ s = 120, *λ* = 0.999807 (forgetting factor) and OBG = 240 (the order of the median filter for the background sequence).

#### 2.3.4. Decision

To decide whether seizure occurs or not, the dimensionless ratio, *r_k_*, is calculated as

(4) $$r_{k}=\frac{FG_{k}}{BG_{k}}.$$

Then, two parameters, threshold, Th_on_, and duration, D_on_, are used. When *r_k_* remains at a given Th_on_ or above for a given D_on_, the signal is decided as a seizure.

### 2.4. Simulation

#### 2.4.1. Supply Voltage Conditions

We have previously reported equivalent circuit models of the frequency-up-converting impact-based piezoelectric energy harvester and its validity [17,22]. We simulated to find the lowest available frequency of external vibrations to support stable power supply voltages to the circuits by using the equivalent circuit model. 

**Figure 7:**
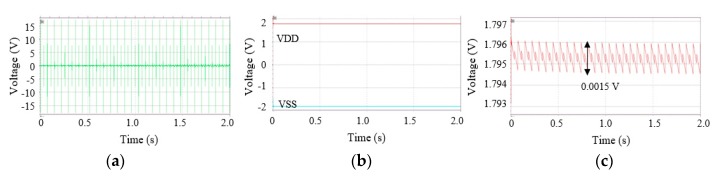
Simulation results in case of a regular vibration, (**a**) harvester voltage outputs, (**b**) a VDD and a negative power supply voltage (VSS) for the neural recording circuits, and (**c**) zoomed-in VDD from (b).

Figure 7 shows simulation results at a regular vibration: 13.5 Hz excitation with 3.5 g acceleration. As shown in Figure 7a, the harvester generated voltage signals regularly twice in one period, 74 ms. Peak values were 17.6 V and 8 V, respectively. Figure 7b shows a VDD and a VSS of the neural recording circuits. As shown in Figure 7c, the VDD was stable at an average of 1.7953 V with a ripple of ±0.05%. In other words, when the harvester shook at 13.5 Hz excitation with 3.5 g acceleration, charged and discharged energy at Cs were well balanced at 1.795V.

**Figure 8:**
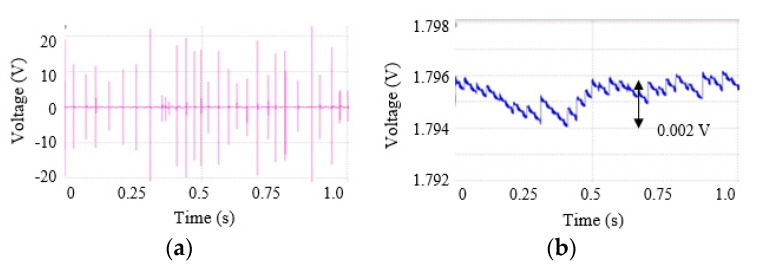
Simulations results in case of irregular vibrations: (**a**) Reference vibration is 13.5 Hz excitation with 3.0 g acceleration, a generated voltage by the harvester, (**b**) VDD of the neural recording circuits.

Figure 8 shows a voltage signal generated by the harvester and a VDD in case of irregular vibrations. The reference vibration period was 74 ms (13.5 Hz) and its variance was ±40%. In other words, the period was in the range of 44.4 ms (22.5 Hz) to 103.6 ms. (9.7 Hz). We assumed that the average acceleration was 3.0 g and the variance of the generated voltage was also ±40%. As a result, the VDD was changed within ±0.006% of 1.795 V.

In comparison with the generated voltage at a regular vibration as shown in Figure 7a, the generated voltages at an irregular vibration as shown in Figure 8a had many different magnitudes and intervals. When an interval between collisions was increased, the reduction of the supply voltage was increased. When the interval was decreased, the supply voltage rose overall. Therefore, the VDD at an irregular vibration had wider variation than at a regular vibration. 

#### 2.4.2. Simulation Method

**Figure 9:**
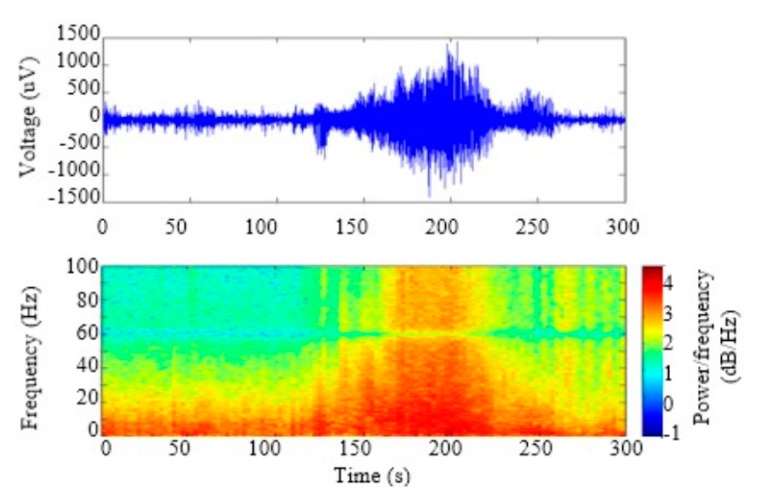
One recorded ictal EEG data and its spectrogram.

We used 15,300-s segments of seizure data and 1510-min segments of non-seizure data from 9 epilepsy patients. Each seizure segment had one seizure event, the duration of which ranged from 30 to 120 s. Figure 9 shows one recorded seizure data and its spectrogram. The seizures start at about 120 s and have a high-amplitude (>300 uV) and high-frequency oscillation (>20 Hz).

**Figure 10:**
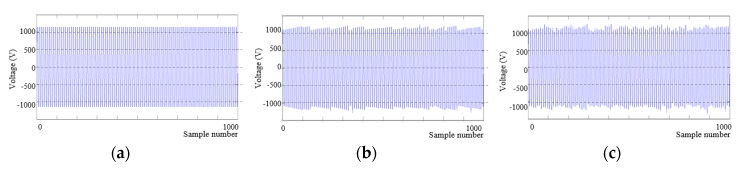
Example explaining the effects of supply voltage variations by using a sine wave. (**a**) A raw sine signal, (**b**) a signal in which distortions caused by supply voltage variations were reflected, and (**c**) a modified signal by adding noise to the signal b.

**Figure 11:**
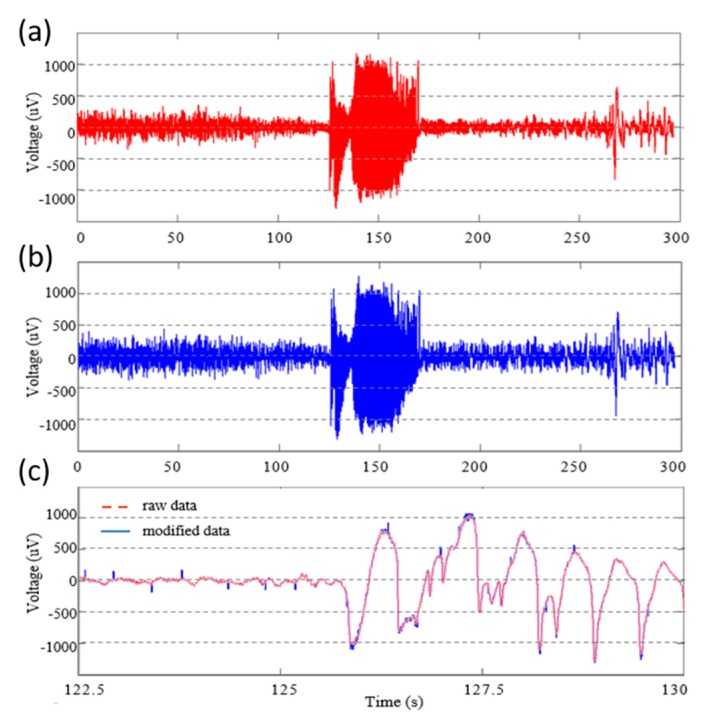
Example explaining the effects of supply voltage variations by using a seizure segment. (**a**) A raw seizure segment, (**b**) data in which distortions caused by supply voltage variations and noise were reflected, and (**c**) zoomed-in data of (**a**,**b**).

We modified recorded data on the basis of the simulation results. Figure 10 shows an example explaining the effects of supply voltage variations by using a sine wave (Figure 10a). A signal in Figure 10b reflected distortions due to irregular supply voltage variations. An envelope of the signal took the form of an irregular ramp. Figure 10c shows a modified signal by adding noise to the data in Figure 10b. Figure 11 shows another example by using a 300-s seizure segment (Figure 11a). Figure 11b shows a modified signal, which distortions and noise were applied to. Figure 11c shows the enlarged signal for easier comparison. The modified signal had irregular spikes. Significant differences between the raw signal and the modified signal occurred mostly when an amplitude of a raw signal was large.

#### 2.4.3. Performance Measures

**Figure A1:**
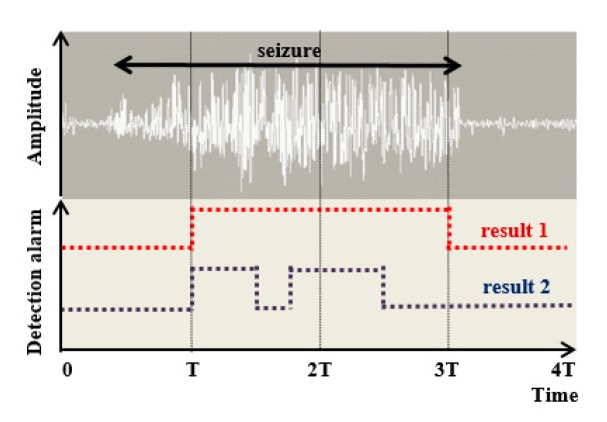
Examples of different detection results during a seizure.

To analyze detection performance, we considered three terms: Detection time, specificity, and sensitivity. Specificity and sensitivity were defined as described by Equations (5) and (6), respectively [23].

(5) $$\mathrm{specificity}=\;\frac{TN}{TN+FP}$$

(6) $$\mathrm{sensitivity}=\;\frac{TP}{TP+FN}$$

where *TN* and *FP* represented the true negatives and false positives, respectively, and *TP* and *FN* represented the true positives and false negatives, respectively. We calculated *FP* by the percentage of time spent under false positive alarms (%*FP_time*) [23] instead of the absolute number of false positives of the false positive rate. Similarly, we calculated TP by the percentage of time spent under the true positive alarms (%*TP_time*) (Appendix A Figure A1). As a result, we measured the performance in terms of detection time, %*FP_time*, and %*TP_time*.

## 3. Results

**Figure 12:**
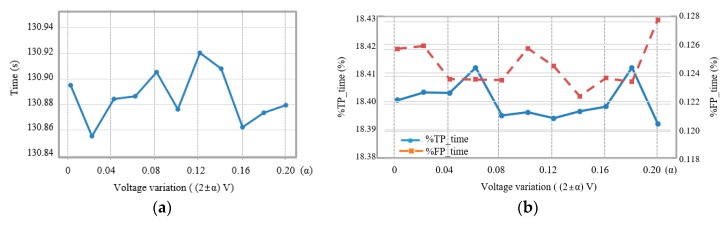
(**a**) Average detection time and (**b**) average %*TP_time* and %*FP_time* according to regular variations of the supply voltage.

Figure 12a shows average detection time values over ±0.2 V regular variations of a 2 V supply voltage as shown in Figure 5a. When the variance of the supply voltage was zero, a seizure was detected at 130.895 s. The seizure detection times were varied from 130.855 s to 130.920 s according to the voltage variances. As a result, the average time difference between with and without regular voltage variances was about 0.05 s. 

Figure 12b shows average %*TP_time* and average %*FP_time*, respectively, when the supply voltage varied regularly in the range of ±10%. When the supply voltage was without variance, %*TP_time* was 18.401% and %*FP_time* was 0.1258%. These results indicated the true positive alarms were raised for an average of 55.2 s over a 300-s seizure segment and the false positive alarms occurred for an average of 0.75 s over a 10-min inter-seizure data. %*TP_time* and %*FP_time* varied within about 0.02% (0.06 s) and about 0.004% (0.024 s), respectively, according to the regular supply voltage variances from 0% to ±10%.

**Figure 13:**
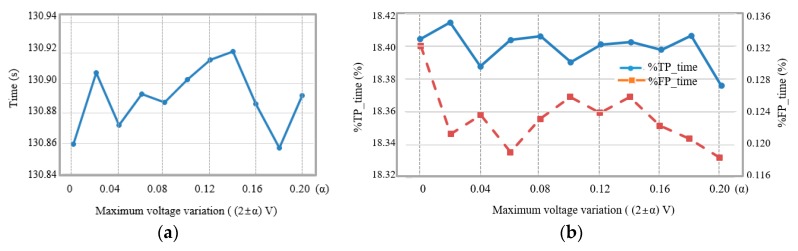
(**a**) Average detection time and (**b**) average %*TP_time* and %*FP_time* according to irregular variations of the supply voltage.

Figure 13a shows average detection time values when the harvester shaken irregularly so that an average voltage was 2 V and maximum voltage variances were in the range of 0 V to 0.2 V at 0.02 V intervals. Seizures were detected at an average of 130.860 s without supply voltage variation. The seizure detection times varied between 130.857 s and 130.921 s in the maximum voltage variation range of ±0.2 V. Consequentially, the average detection time was changed within 0.07 s when the harvester shaken irregularly.

Figure 13b shows average %*TP_time* and average %*FP_time*, respectively, when the supply voltage was varied irregularly in the range of ±10%. When the supply voltage was without irregular variance, %*TP_time* was 18.404% and %*FP_time* was 0.132%. %*TP_time* and %*FP_time* varied within about 0.029% (0.087 s) and about 0.014% (0.084 s), respectively, according to the irregular supply voltage variances from 0% to ±10%.

## 4. Discussion

We found that the average time differences between with and without voltage variances were about 0.05 s under regular vibrations and about 0.07 s under irregular vibrations, respectively. They were considered to be tolerable when considering that Osorio et al. [18] showed the mean delay from the electrographic onset to automated detection was varied from −0.34 s to 3.9 s according to detection parameters under an ideal voltage source. In addition, %*TP_time* and %*FP_time* were nearly impervious to ±10% regular and irregular variations of the supply voltage. 

The variance of the supply voltage could cause non-linear distortions and spikes with the same frequency as the variance of the supply voltage. Most of the seizure detection algorithms, including the GOFA, could distinguish between seizures and spikes. The GOFA filtered out spikes primarily using a median filter and then decided on a seizure when a state transition from non-seizure to seizure occurred for at least a threshold, D_on_ [18]. Furthermore, they have usually concentrated on spectral analysis and entropy. Therefore, non-linear distortions and spikes caused by the variance of the supply voltage within ±10% affected performance a little.

The proposed frequency-up-converting impacted-based piezoelectric energy harvester may still be heavy to be implantable. But as research continues, it is expected to shrink to a reasonable size in the near future. In addition, we were aware that we did not test the harvester and circuits together in vivo. However, we used each model that reflected its behavior characteristics under implanted conditions. Therefore, our models and study results could help the design of energy harvesters, power transfer circuits, implantable hardware devices, and algorithms for implantable devices.

## 5. Conclusions

We estimated the performance of a seizure detection algorithm with the neural signal recording circuits powered by the harvester. We tested and analyzed the piezoelectric energy harvester and its load circuits: The power conversion circuits and the neural recording circuit. To operate the neural signal recording circuits under the lower supply voltage variation of ±10%, the frequency-up-converting impact-based piezoelectric energy harvester has to shake regularly at a rate of at least 13.5 Hz with 3.5 g acceleration, or irregularly at a frequency range of 9.7 to 22.5 Hz with a maximum acceleration of 4 g. By using equivalent circuits for the harvester and circuits, we simulated them under the operating conditions. According to simulation results, seizure and non-seizure data were modified as they were processed under supply voltage variance ranging from 0% to ±10%. The seizure detection algorithm GOFA was used to estimate performance variation about these data. The onset detection time, %*TP_time*, and %*FP_time* were changed to less than 0.1s, 0.03% and 0.02%, respectively. The results showed that a supply voltage variance within ±10% could be acceptable to a seizure detection algorithm. Therefore, it shows that energy harvesters have the potential to become a reliable power source for implantable devices and reduce replacement of implantable devices due to dead batteries.

## Appendix A

Figure A1 shows the concept of %*TP_time*. One seizure segment was tested by two different detection algorithms. Detection results were displayed. If *TP* was to be measured according to the absolute number of true positive alarms, result 2 would appear to have the better performance. However, if *TP* was to be measured in terms of %*TP_time*, result 1 and result 2 had 50% and 31.25% %*TP_time*, respectively. Result 1 was therefore considered to have the better performance than result 2 [24].
